# Cellular Effects of Pyocyanin, a Secreted Virulence Factor of *Pseudomonas aeruginosa*

**DOI:** 10.3390/toxins8080236

**Published:** 2016-08-09

**Authors:** Susan Hall, Catherine McDermott, Shailendra Anoopkumar-Dukie, Amelia J. McFarland, Amanda Forbes, Anthony V. Perkins, Andrew K. Davey, Russ Chess-Williams, Milton J. Kiefel, Devinder Arora, Gary D. Grant

**Affiliations:** 1 Menzies Health Institute Queensland, Griffith University, Queensland 4222, Australia; s.hall@griffith.edu.au (S.H.); s.dukie@griffith.edu.au (S.A.-D.); a.mcfarland@griffith.edu.au (A.J.M.); a.perkins@griffith.edu.au (A.V.P.); a.davey@griffith.edu.au (A.K.D.); d.arora@griffith.edu.au (D.A.); 2 School of Pharmacy, Griffith University, Queensland 4222, Australia; 3 Centre for Urology Research, Faculty of Health Sciences and Medicine, Bond University, Queensland 4226, Australia; camcderm@bond.edu.au (C.M.); aforbes@bond.edu.au (A.F.); rchesswi@bond.edu.au (R.C.-W.); 4 School of Medical Science, Griffith University, Queensland 4222, Australia; 5 Institute for Glycomics, Griffith University, Queensland 4222, Australia; m.kiefel@griffith.edu.au

**Keywords:** inflammation, oxidative stress, *Pseudomonas aeruginosa*, pyocyanin, virulence factor

## Abstract

Pyocyanin has recently emerged as an important virulence factor produced by *Pseudomonas aeruginosa*. The redox-active tricyclic zwitterion has been shown to have a number of potential effects on various organ systems in vitro, including the respiratory, cardiovascular, urological, and central nervous systems. It has been shown that a large number of the effects to these systems are via the formation of reactive oxygen species. The limitations of studies are, to date, focused on the localized effect of the release of pyocyanin (PCN). It has been postulated that, given its chemical properties, PCN is able to readily cross biological membranes, however studies have yet to be undertaken to evaluate this effect. This review highlights the possible manifestations of PCN exposure; however, most studies to date are in vitro. Further high quality in vivo studies are needed to fully assess the physiological manifestations of PCN exposure on the various body systems.

## 1. Introduction

*Pseudomonas aeruginosa* is a Gram-negative bacterium responsible for severe nosocomial infections in a number of body systems including the respiratory tract, the vascular system, the urinary tract, and the central nervous system (CNS) [1,2,3,4,5,6,7]. It has been well documented that *P. aeruginosa* produces a number of secreted virulence factors known as phenazines. One such compound is pyocyanin (PCN) [8] which has been widely studied to date and is emerging as a virulence factor of interest given the antimicrobial resistant, chronic nature of pseudomonal infections [9].

**Figure 1:**
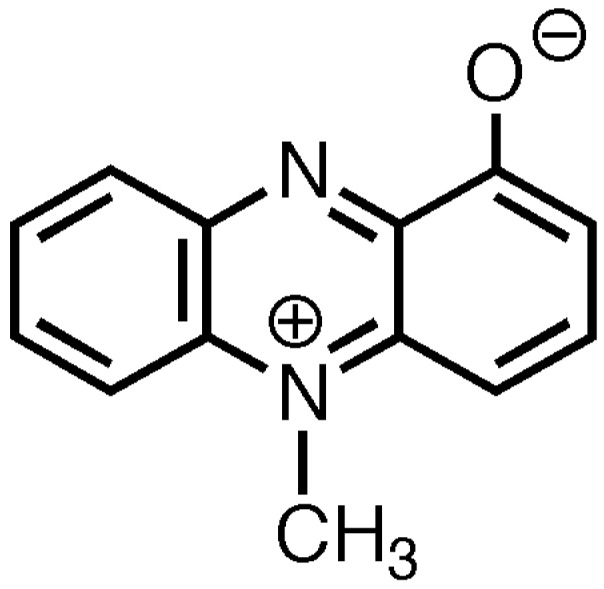
Chemical structure of PCN.

PCN is a nitrogen-containing aromatic compound belonging to the tricyclic phenazine class of compounds [8,10,11,12,13,14,15] as seen in Figure 1. PCN is a zwitterion [8,14,16] containing a phenol group, giving it weak acidic characteristics (pKa of 4.9) [10,12,17,18]. At physiological pH, PCN exists in its ionized, neutral state (blue) and in its protonated, charged form (red) when in an acidic environment [8,16,18]. The low molecular weight and zwitterionic properties of PCN are believed to permit the toxin to easily permeate cell membranes [8,14,16]. Despite this reported ability to cross biological membranes, the presence of PCN in systemic circulation has yet to be reported. In light of this, reports on the levels of PCN are limited to the compartments directly associated with infection. PCN is secreted by *P. aeruginosa* into the local environment by a type II secretion system [19]. Significant levels of PCN have been detected in sputum sol (up to 130 μM), ear secretions (up to 2.7 μM), wounds (up to 8.1 μM), and urine following chronic infection by *P. aeruginosa* [20,21,22]. These values indicate the possible relevance of this virulence factor in the pathophysiology of pseudomonal infections and possible toxic effects in the biological systems where the infection is located. Further studies are needed to evaluate the diffusibility of this molecule in both in vitro and in vivo studies.

To date, numerous studies have shown the potential importance of PCN in the virulence and pathogenicity of pseudomonal infections and its potential toxic effects [14,23,24]. PCN has been shown to have numerous antagonistic effects on the host, both in vivo and in vitro, including pro-inflammatory and free radical effects resulting in cellular damage and death [23,25,26,27]. Given the high incidence of chronic colonization with *P. aeruginosa* in cystic fibrosis (CF), the majority of research on PCN to date has primarily been focused on its effect on human airway. However, recently there have been a number of studies demonstrating the broader consequences of PCN exposure, especially in the urinary tract [27,28], the cardiovascular system [29], and the CNS [30,31]. In light of this, the focus of the review is to highlight the diverse cellular effects of PCN in vitro, in vivo, and in humans. This review highlights the importance of PCN in not only the pathophysiology of pseudomonal lung disease but also in the pseudomonal infections of other organ systems.

## 2. Role of Oxidative Stress in Pyocyanin’s Toxicity

**Figure 2:**
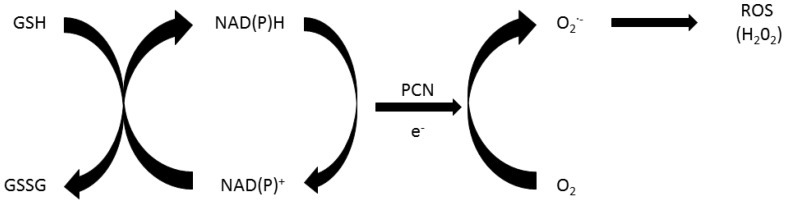
Mechanism of PCN-induced oxidative stress [39].

Oxidative stress is a major contributing factor to the cytotoxicity displayed by PCN [32,33], a reversible redox-active compound with its effects seen in Figure 2 below [8,12,17,18,21,34]. PCN’s induction of oxidative stress is, at least in part, due to its ability to increase intracellular levels of reactive oxygen species (ROS), in particular superoxide (O_2_·^−^) [10,11,12,17,25,34,35] and hydrogen peroxide (H_2_O_2_) [25,36]. These increases are mediated by dismutase and under aerobic conditions, H_2_O_2_ and O_2_·^−^ and are formed by cyclic non-enzymatic reduction by NAD(P)H [12,17,34] with PCN accepting electrons from NAD(P)H [10,11,12]. The intracellular ROS formed after PCN exposure cause free radical damage resulting in oxidative damage to components of the cell cycle, as well as direct damage to DNA [17,37], NAD(P)H depletion and enzyme inhibition [17] with the main target, the mitochondria of cells [38].

In further support of the integral role of oxidative stress in PCN’s ability to induce cytotoxicity, it has been demonstrated that strains of *P. aeruginosa* that overproduce PCN produce greater levels of oxidative stress, subsequent cell lysis, and a significant increase in extracellular DNA (eDNA) [40]. To further enhance the virulence of *P. aeruginosa*, through an oxidative stress-dependent mechanism, PCN has then been shown to intercalate with eDNA to promote cell-to-cell interactions between *P. aeruginosa* cells by influencing their cell surface properties and physicochemical interactions. It has, therefore, been suggested that PCN may also contribute to biofilm formation by the promotion of eDNA [40]. This study highlights the fact that not only does PCN-induced oxidative stress result in direct cellular damage and death, but also in the persistence of infections caused by *P. aeruginosa* via the above mechanism.

Infection by *P. aeruginosa*, and subsequent secretion of PCN, has been shown to have a dramatic effect on the cellular protective mechanisms of the enzymes superoxide dismutase (SOD), catalase (CAT), and glutathione peroxidise (GSH-Px) [41]. When cells are exposed to conditions of increased oxidative stress, such as exposure to PCN, their SOD and CAT activities increase to compensate [38]; however, it has been shown that SOD2 transcript levels decrease 8.5-fold in the presence of PCN [42], indicating further studies are required. SOD exists in two intracellular forms, converting O_2_·^−^ to H_2_O_2_, thereby acting as the first protective mechanism against PCN induced oxidative stress [43]. Subsequently, the H_2_O_2_ generated from both PCN and SOD, is then in turn partially removed by the enzyme CAT [44] or by the redox action of cellular thiol compounds, such as glutathione (GSH) and thioredoxin [45,46]. PCN exposure decreases the activity of CAT, a peroxisomal enzyme and an important component of the antioxidant defense system [38]. It has been postulated that this ability of PCN is partially due to its consumption of catalase-associated NADPH resulting in increased production of O_2_·^−^ and H_2_O_2_ [10,38] and a subsequent cycle resulting in cellular damage and death.

A second protective mechanism against free radical damage is through the redox action of the thiol compounds such as GSH [45], whereby free radicals are removed via a non-enzymatic reduction with GSH. Conversely, the removal of hydroperoxides requires enzymatic catalysis by GSH-Px. Both of these reactions lead to the generation of glutathione disulfide (GSSG, or oxidized glutathione), which is then in turn reduced back to GSH by glutathione reductase [47]. PCN has been suggested to directly oxidize GSH, thereby promoting PCN-mediated ROS formation and subsequent oxidative stress [11]. Furthermore, PCN produces a concentration-dependent decrease in GSH levels through its ability to increase in H_2_O_2_ levels, as described earlier [11]. During this process, PCN returns to its reduced form, again capable of creating ROS [11]. Treatment with exogenous GSH or GSH precursors, such as N-acetylcysteine (NAC), is known to provide protection against PCN-induced oxidative injury [48]. This further implicates PCN exposure in cellular damage resulting from oxidative stress.

Recently, the effects of PCN-induced free radical formation on various cell signaling pathways have been assessed in a number of studies. It has been reported that PCN induced the oxidative stress-dependent induction of MUC2 and MUC5AC, both genes encoding for mucin secretion, through a mechanism involving epidermal growth factor receptor/extracellular signal-regulated kinase (EGFR/ERK) activation. This resulted in increased mucous secretion in the respiratory tract further aiding in the colonization of the airways by *P. aeruginosa* [39]. The underlying EGFR signaling mechanism in this experimental model remains to be fully elucidated. However, it has been proposed that EGFR may be either activated directly through ROS, pro-inflammatory cytokines, or by EGFR ligands [39]. A subsequent study found that PCN rapidly increased ERK1/2 phosphorylation however it was shown PCN-induced cytotoxicity was independent of c-Jun N-terminal kinases (JNK) and p38 mitogen-activated protein kinases (MAPK). These results indicate that PCN-induced oxidative stress is independent of ERK1/2 signaling, suggesting that further studies are needed to elucidate the effects of PCN on signaling pathways [49].

The extensive studies on the free radical effects of PCN show the relevance and importance of this property to the virulence and pathogenicity of *P. aeruginosa*. Further studies are needed, however, to fully elucidate the cell signaling pathways involved in these effects.

## 3. Manifestations of PCN Exposure

Studies have been undertaken assessing the impact of PCN on respiratory, urological, neurological, cardiovascular, and hepatic models, both in vitro and in vivo [27,28,29,30,31,33,48,50,51], with the majority of these studies undertaken in in vitro systems. These studies have highlighted the diverse range of effects that PCN has on the host, including pro-inflammatory [11,12,33] and immunomodulating properties [33]. Further to these effects, the presence of PCN in cellular systems places them under increased oxidative stress, resulting in cellular death [12].

### 3.1. Inflammatory Effects of PCN

Numerous studies have investigated the effects of PCN on the host immune function in in vivo and in vitro studies. However, to date, no studies have assessed the inflammatory effects in humans [26,37,52]. This review will outline the in vivo and in vitro inflammatory effects below.

In vivo studies have predominantly investigated immunological effects in the respiratory tract of animals and will be discussed in further detail below. PCN has, however, been shown to alter the expression and release of a number of cytokines in vitro [26,37,52]. PCN has been shown to inhibit the release of interleukin (IL)-2 [37,52,53] and decreases the expression of IL-2 receptors on T-cells [34,53]. This subsequently leads to the decrease in the secretion of immunoglobulin by B-lymphocytes [37] and inhibition of lymphocyte proliferation [21,32,34], resulting in a decreased immune response and, thereby, potentially protecting *P. aeruginosa* from the immune system. PCN concentrations as low as 40 µM (0.08 mg/mL) have been shown to be toxic to lymphocytes, which are consistent with reported physiological concentrations [54].

In addition to effects on lymphocytes, PCN also has toxic effects on neutrophils, predominantly through apoptotic pathways [55]. Neutrophils play an important role in the pathogenesis of CF and possibly other respiratory disorders with increased numbers observed in the tissue of lungs [56] with the influx of neutrophils resulting in an increase in inflammation [50]. PCN attracts neutrophils through the production of two neutrophil chemotaxins, IL-8 and leukotriene B_4_ (LTB_4_) [52], and induces a rapid and selective apoptosis of neutrophils at concentrations that are found in the sputum of CF patients [57]. PCN exposure has been shown to increase in IL-8 levels [52], through a signal transduction pathway [26]. This pathway includes oxidants, protein tyrosine kinases (PTKs), and MAPKs [26]. Recent studies investigated the signaling pathways further and found PCN increased IL-8 secretion in a time and concentration-dependent manner and suggest the phosphokinase C (PKC), p38, and ERK MAPKs, and nuclear factor-kappa B (NF-κB) are involved in mediating the increases [58,59]. Neutrophil apoptosis [23,37,55,60,61] and impairment of neutrophil-mediated host defenses are other effects PCN plays on neutrophils [61]. Apoptosis after exposure to PCN is time- and concentration-dependent, occurring at concentrations of 10 µM (2.11 µg/mL) and above. Concentrations of 50 µM (10.55 µg/mL) PCN induce a 10-fold increase in neutrophil apoptosis at 5 h [60]. This effect is thought to be mediated through the increase of reactive oxygen intermediates and a decrease in intracellular cyclic AMP (cAMP) concentration [60]. A recent study identified that one such potential mechanism involving PCN induction of mitochondrial ROS which subsequently induces neutrophil death and apoptosis via mitochondrial acid sphingomyelinase [62].

Neutrophils are additionally responsible for the release of large amounts of elastase and other proteases [56,63], as well as various oxidants and enzymes [64], all contributing factors to the progressive lung destruction experienced by CF and bronchiectasis patients [63,65]. The proteases released by neutrophils overwhelm the local host defenses, including α-1 antitrypsin and leukocyte protease inhibitor, important in protecting tissue from proteases released from inflammatory cells, leading to increased risk of chronic infection [63,65]. To further complicate the issue, PCN inactivates α1-antitrypsin [36], resulting in an imbalance of protease-antiprotease activity in the airways of CF and bronchiectasis patients [36], and ultimately results in increased levels of human neutrophil elastase [66]. Human neutrophil elastase is responsible for the breakdown of lung tissue and ultimately contributes to the pathogenesis of CF [36] and other respiratory disorders. Neutrophils also play an important role later in the inflammatory response with their breakdown producing large amounts of high molecular weight DNA. High molecular weight DNA increases the viscosity of the mucous and ultimately contributes to decreased mucociliary clearance [67]. Under inflammatory conditions associated with the *P. aeruginosa* infection, stimulated neutrophils secrete myeloperoxidase (MPO) and H_2_O_2_, both of which are essential components of a peroxidase system and are known causes of oxidative stress [12]. Bianchi et al. (2008) showed that PCN impaired macrophage engulfment of apoptotic cells as a result of intracellular ROS generation and modulation of small GTPase signaling [68]. This identifies a novel and potentially important mechanism by which pathogens could disrupt efficient clearance of inflammatory cells, increasing host tissue injury [68].

PCN has been shown to exert various other effects on inflammation including its ability to decrease Regulated upon Activation, Normal T Expressed and Secreted (RANTES) [52], an important chemokine involved in the chemoattraction of macrophages and CD4+ memory T-cells [69]. This may alter the normal immune response, leading to reduced clearance of *P. aeruginosa* and, ultimately, further contributing to tissue damage. Interestingly, PCN has been shown to have little direct effect on inducing monokines, including IL-1 and tumor necrosis factor (TNF) by monocytes, but significantly enhances their lipopolysaccharide (LPS)-stimulated production [54]. This is of particular importance in *P. aeruginosa* infection, due to the bacterium’s ability to produce LPS. These effects lead to the suppression of defense mechanisms and enhance harmful inflammatory reactions of the host during infection with *P. aeruginosa* [54], potentially resulting in increased virulence and colonization.

### 3.2. The Effects of PCN on the Respiratory System

The respiratory effects of PCN have been extensively studied due to the high prevalence of pseudomonal infections in respiratory conditions such as CF and bronchiectasis [21]. The damage to the respiratory system can be primarily attributed to the free radical and inflammatory effects of PCN outlined above. Furthermore, significant concentrations of PCN have been detected in the sputum sol of colonized patients, with concentrations up to 130 µM [21]. The respiratory effects of PCN have been evaluated in in vitro, in vivo and human studies and are outlined below.

A limited number of human studies have evaluated the effects of PCN on the respiratory tract [21,70]. The sol phase isolated from patients with colonization with *P. aeruginosa* with detectable concentrations of PCN was shown to significantly slow ciliary beating in all nine cases [21]. Furthermore, in eight of the nine cases in this study, ciliary dyskinesia, ciliary stasis, and epithelial disruption occurred, suggesting a concentration-dependent mechanism may be responsible [21]. A second human study investigated the effects of sputum PCN concentration on lung function in 48 CF patients [70]. An inverse correlation was observed between sputum PCN concentration and lung function providing further evidence of the destructive nature of PCN [70].

In addition to the human studies, a limited number of in vivo studies have been conducted evaluating the effects of PCN on the respiratory tract [23,50,71,72]. PCN exposure has been shown to induce several negative changes in the respiratory tract predominantly through its pro-inflammatory and pro-oxidant effects [23,50,71,72]. Several studies have shown PCN exposure to induce neutrophil influx into the lungs after local instillation into the respiratory tract suggestive that PCN plays a role in the mediation of goblet cell hyperplasia and pneumonia development [23,50,71,72]. This was shown to occur as early as 3 h and persist for 24 h post-PCN exposure and occurred in a concentration-dependent manner [23,71]. Increases in murine neutrophil chemokines keratinocyte chemoattractant (KC), macrophage inflammatory protein (MIP-2), and intracellular adhesion molecule 1 (ICAM-1), all of which play a role in the migration of neutrophils, were observed after animals were treated into the airways with 100 µM of PCN [71].

A recent study suggests that PCN suppresses FoxA2, a transcriptional repressor of goblet cell hyperplasia and mucous production, via the upregulation of Stat6 and EGFR [72]. It is suggested that this may occur through either a pro-inflammatory response mediated through a Th2 response and Stat6 signaling or the production of ROS and MEK1/2 and ERK1/2 signaling, both known to downregulate the expression of FoxA2 [72]. Furthermore, significant changes to tracheal mucous velocity have been observed with relatively low doses of PCN (600 ng) occurring 3 h post-PCN exposure and no recovery observed [73]. These studies suggest that PCN plays an important role in the production of mucous in the lungs of infected patients.

The majority of studies investigating the effects of PCN in respiratory cells have been done in in vitro models and have evaluated the effects of PCN on oxidative stress and inflammatory-mediated damage. One such study showed that when both A549 and primary NHBE respiratory cells were exposed to ≥5 µM concentrations of PCN over at least 12 h, the activity of catalase decreased significantly [74]. Similar to SOD, CAT transcription appears to also be adversely affected by PCN-mediated ROS formation. In the presence of PCN, CAT mRNA levels were decreased in human bronchial H292 cells [42], suggesting PCN may negatively affect both expression and function of cellular antioxidant defense mechanisms. Additionally, Muller et al. (2006) found that exposure of A549 cells to PCN resulted in the formation of H_2_O_2_ in a dose-dependent manner and that the senescence-inducing effect of PCN could be overcome in the presence of glutathione at concentrations of 0.5 mM above [34].

Recently, oxidative stress has been causally linked to PCN-induced toxicity in human respiratory A549 cells [48]. It was found that supplementation of the antioxidant NAC prior to PCN exposure protected cells from oxidative stress and cytotoxicity. PCN is known to induce oxidative stress-dependent mucin hypersecretion in bronchial epithelial cells [42]. Overproduction of mucin glycoproteins prevents airway clearance and provides a favorable environment for *P. aeruginosa* colonization in CF airways [39].

Investigations into the effects of PCN on pro-inflammatory mediators and the chemotaxis of polymorphonuclear (PMN) cells have been undertaken [50]. After 24 hours of PCN exposure, IL-8 concentrations produced by alveolar macrophages significantly increased suggesting a pro-inflammatory response [50]. Furthermore, chemotactic activity was observed in PMN cells exposed to PCN for 24 h [50]. These results suggested that the observed inflammatory response post-PCN exposure was mediated through the production of chemotactic factors [50]. A second study investigated the effects of 48 h PCN exposure on surface ICAM-1, finding that exposure increased its expression in a concentration-dependent mechanism [71]. Furthermore, it was shown that PCN synergizes with IL-1β and tumor necrosis factor alpha (TNF-α) in increasing ICAM-1 however this effect can be blocked by the addition of NAC in a concentration-dependent manner [71].

The studies outlined above highlight the importance of the free radical effects PCN produces and highlights the importance of this mechanism as major cause of the pathogenicity of this compound.

### 3.3. The Effects of PCN on the Urological Systems

*P. aeruginosa* is implicated in a large percentage of nosocomial urinary tract infections, particularly in patients with urethral catheters, and is commonly associated with high levels of antibiotic resistance [2]. Chronic and recurrent urinary tract infections are, therefore, common; a characteristic attributed to their ability to form biofilms on the surface of urinary catheters, adhere to the urothelium and secrete an array of virulence factors. Although no literature to date specifically assess levels of PCN in the bladder, up to 39 µM PCN has been detected in vitro from urinary isolates and Al-Ani et al. (1986) reported that urinary pseudomonal isolates produced more PCN than isolates form other clinical specimens tested [75]. To date studies on the effects of PCN on the urological system have only been undertaken in vitro and are outlined below.

A recent study by McDermott et al. (2012) was the first to assess the effects of PCN on the urothelial cells. They reported that PCN significantly reduced RT4 urothelial cell viability at concentrations of 25 µM and greater [28]. Furthermore PCN induced cellular senescence in RT4 urothelial cells at concentrations of 25–50 µM, demonstrated by increased SA-β-gal expression coinciding with observed changes in morphological characteristics. Induction of cellular senescence by PCN inhibits the ability of wound tissue to repair [76], suggesting a possible role for senescence in preventing urothelial repair during urinary tract infection. Reduction in urothelial barrier function as a result of PCN exposure may, therefore, contribute to recurrent infection. In contrast, 100 µM PCN showed no significant effect on SA-β-gal expression, however, increased caspase-3 activity suggesting cellular apoptosis [28]. Therefore, it is likely that the effect of PCN on urothelial function is concentration-dependent.

This study also provided evidence that PCN reduced baseline and stimulated adenosine triphosphate (ATP) release [28]. This suggests that despite the role of ATP in mediating perception of pain, pain associated with *P. aeruginosa* urinary tract infections may not be associated with enhanced urothelial ATP release.

A second study on the effects of PCN on the urinary tract investigated the inflammatory effects. It was found that PCN increased prostaglandin E_2_ (PGE_2_) production in RT4 urothelial cells in a concentration-dependent manner, with statistically significant increases observed at 100 μM. Furthermore, it was found that 100 μM of PCN increased IL-6 levels in the same cell line [27]. This study suggested that PCN-induced increases in IL-6 in urothelial cells may contribute to local urinary tract symptoms and highlights the possible inflammatory contribution of PCN in urinary tract infections caused by *P. aeruginosa* [27].

### 3.4. The Effects of PCN on the Central Nervous System

Infection of the CNS with *P. aeruginosa*, though rare, is associated with high mortality. There is currently limited evidence to support mechanisms underlying this toxicity, with all available studies currently using in vitro models. A preliminary study by McFarland et al. (2012) was the first to determine the effect of PCN in a neurological model, identifying that PCN treatment induced toxicity in 1321N1 astrocytoma cells, and correlated with the formation of acidic vesicular organelles (AVOs) [30]. This finding is consistent with induction of macroautophagy—a conserved lysosomal degradation pathway involving the recycling of cellular proteins and organelles. The addition of 3-methyladenine, an inhibitor of macroautophagy through inhibition of class III phosphoinositide, provided complete protection from PCN toxicity, further suggesting that PCN may interfere with the inherent autophagic processes of these cells. Furthermore, McFarland et al. (2012) identified that whilst PCN still induced oxidative stress, apoptosis, and cellular senescence in astrocytoma cells, these appeared to occur secondary to AVO formation [30].

A second follow-up study, comparing the effects of PCN on both 1321N1 astrocytoma cells and SH-SY5Y neuroblastoma cells, found SH-SY5Y cells to be significantly more resistant to the toxic effects of PCN [31]. Of particular interest, it was shown that 3-methyladenine, previously shown to be protective against PCN-induced toxicity in 1321N1 astrocytoma cells, had a paradoxical effect in SH-SY5Y neuroblastoma cells, decreasing the cells’ resistance to PCN toxicity. Additionally, it was observed that PCN exposure in both cell lines resulted in similar increases in inflammatory mediators as with other in vitro models [27]. Significant PCN-induced increases in IL-8, PGE_2_ and were observed with both cell lines, implicating PCN-induced inflammation as a possible contributing factor to the pathophysiology of CNS pseudomonal infections. This study established that the toxic effects of PCN in these cell lines was independent of oxidative stress, hence further supporting the role of autophagy-mediated death in PCN-treated 1321N1 and SH-SY5Y cells [31]. Further investigation is required to fully establish the mechanisms of PCN in in vitro neurotoxicity and, in particular, in vivo studies are needed.

### 3.5. The Effects of PCN on the Vascular System

Few studies have evaluated the effects of PCN on the vascular system to date, and those available are limited to in vitro studies [29,77,78]. However, *P. aeruginosa* is a rare cause of infective endocarditis and is seen primarily in injection drug users and patients undergoing cardiac procedures [3,4,5,6]. Therefore PCN potentially has an effect on the function of the vascular system in these patients. Additionally, given the unconfirmed status of diffusibility of PCN into systemic circulation from local infections, it is possible that vascular effects may be observed in patients, particularly those with prolonged exposure, such as CF. Vascular manifestations may also arise from *P. aeruginosa* sepsis, a life-threatening systemic infection that is becoming more prevalent in society [79]. PCN, at physiologically relevant concentrations, has been shown to inhibit vasorelaxation of porcine coronary arteries via both nitric oxide-dependent [29,77] and -independent mechanisms [29]. A recent study has shown that CF patients experience endothelial dysfunction [78] which may be further exacerbated through exposure to PCN. Furthermore, these effects in combination with the effects of PCN on NO suggest that exposure may relate to an increased risk of hypertension, however, further in vivo and human studies are needed to fully investigate this possible effect.

### 3.6. The Effects of PCN on the Hepatic System

To date, only a small number of in vivo studies have been undertaken assessing the effects of PCN on hepatic function [33,51]. PCN was shown to significantly alter the morphology of liver sinusoidal endothelial cells (LSEC) including a reduction in the porosity of these cells [33]. Protection from these changes in morphology was however achieved with the addition of CAT, suggesting a free radical-mediated mechanism of toxicity [33]. A subsequent study however further investigated the mechanism by which PCN elicited its toxic response and found it to be independent of free radical damage suggesting a novel mechanism of PCN-induced pathogenesis in the liver [51]. The effects of PCN on the liver may result, clinically, in changes to the metabolism of hepatically-cleared drugs, posing potentially serious implications for patients. These effects, however, have not been studied, but would provide useful information if studied in the future.

### 3.7. Endocrine Effects of PCN

Limited in vitro studies have been undertaken assessing the effects of PCN on the development of diabetes to date. PCN has been shown to be able to deplete cellular ATP and inhibit aconitase, a key component of the Krebs cycle [38]. Aconitase has been long shown to play a role in the development of diabetes, with a decrease in its activity seen in both alloxan and genetic diabetes [80,81,82,83]. Given the high incidence of both diabetes and infection with PCN-producing *P. aeruingona* organisms in CF, this mechanism may provide an insight into the associated pathophysiology, however, further studies are needed to confirm these effects. A second potential mechanism by which PCN may impact on the development of diabetes, in CF patients in particular, is through oxidative stress pathways. PCN, as outlined previously, is a highly redox-active compound that causes many of its cytotoxic effects through oxidative stress. Studies have shown that oxidative stress, in particular superoxide, may play an important role in the onset of diabetes and have been reviewed extensively in the past (reviewed in [84]). Given the ability of PCN to generate superoxide [85] it is possible that, in chronic exposure, that PCN may contribute to the onset of diabetes via this mechanism. A third possible mechanism by which PCN may contribute to diabetes associated with CF is through its inactivation of V-ATPase. V-ATPase has been shown to have numerous biologically functions and 1 subunit has been shown to be of importance in the release of insulin from pancreatic β-cells [86]. PCN inhibits V-ATPase by the generation of the ROS, predominantly H_2_O_2_ and is directly correlated to its concentration. Therefore, increasing levels of PCN caused a corresponding decrease in V-ATPase activity in a concentration-dependent manner [37,87]. Given the potential effects of V-ATPase, further studies into the effects of PCN on specific isoforms of the enzymes are warranted. The possible mechanisms of PCN action in the pathogenesis of diabetes in susceptible patients, as outlined above are plausible. However, further studies are needed to explore these effects.

### 3.8. Role of PCN in Normal Monitored Cellular Activities

In vivo studies, using *Caenorhabditis elegans* as a model, have shown phenazines produced by *P. aeruginosa* to mediate its toxic effects through the generation of ROS [88]. A further study investigating the cellular responses of *C. elegans* to pathogenic *P. aeruginosa*, found that surveillance pathways controlling core cellular activities of the organism allow detection of pathogens with the capability to produce virulence factors and undermine vital host functions [89]. This, then, resulted in an alteration of behavior, namely pathogen-avoidance behaviour [89]. Disruption to the core cellular processes associated with aversion behavior were attributed to involvement of the JNK MAPK pathway triggered by an intracellular stress response [89]. Given PCN’s nature to induce mitochondrial oxidative stress, further investigations into its mitochondrial effects have been described [90,91]. PCN was shown to disturb cellular responses in *C. elegans* that resulted in aversion behavior and was found to be mediated through a mitochondrial pathway resulting in unfolded protein responses [90,91]. These studies further suggest that PCN elicits its toxic cellular effects through the production of ROS, particularly targeting the mitochondria of cells and may be a result of protein unfolding when exposed to PCN.

## 4. Future Directions

As highlighted in the studies outlined above, PCN has been shown to impact on the cellular function of many body systems. However, these are predominantly limited to in vitro studies. These studies have suggested that PCN elicits its toxic effects through ROS and inflammatory mediated pathways. This provides us with the basic mechanisms of cellular toxicity associated with PCN exposure and highlights possible pathways to target in the search for therapies to minimize toxicities associated with *P. aeruginosa* infection. Further studies assessing the observed effects are warranted in vivo and in humans, particularly given the studies suggesting that aversion behavior exists in *C. elegans* exposed to PCN. To date very few in vivo studies have investigated the effects of PCN, however, within the limited number of studies undertaken significant cellular damage was reported. Given the highly toxic nature of PCN, future studies investigating strategies to minimize cellular damage associated with PCN exposure should be investigated. Such strategies include modifying either the production of PCN by *P. aeruginosa* or by preventing the toxic effects using pharmacological modulators. To date, there have been a small number of studies assessing the effects of pharmacological agents on the production of PCN from *P. aeruginosa* in vitro. However, these have not been assessed in vivo [92,93,94,95,96,97,98]. Furthermore, numerous compounds with antioxidant properties have been assessed for their protective properties in vitro, however these have not been evaluated for their effectiveness in in vivo studies [48]. Both of these strategies have provided promising evidence to suggest that they may be beneficial in protecting against PCN-induced cellular toxicity.

## 5. Conclusions

Numerous studies have been undertaken assessing the cellular effects of PCN exposure in various body systems, both in vitro and in vivo. A diverse range of toxic effects have been observed in animals and cells exposed to PCN which have been shown to be predominantly mediated by free radical and pro-inflammatory cytokine production. Given the nature of pseudomonal infections, the effects of PCN on the respiratory tract are the most widely studied phenomenon. Recent in vitro studies have, however, shown that PCN has deleterious effects on cells in numerous other body systems, including the CNS, the urolological and the vascular system, with mechanisms other than oxidative stress implicated in a number of circumstances. In light of this, further studies investigating these effects in vivo and, ultimately, in a human population infected with PCN-producing *P. aeruginosa* are warranted to assess the impact of this exposure, physiologically.
